# An ImageJ macro tool for OCTA-based quantitative analysis of Myopic Choroidal neovascularization

**DOI:** 10.1371/journal.pone.0283929

**Published:** 2023-04-21

**Authors:** Aadit Deshpande, Sundaresan Raman, Amber Dubey, Pradeep Susvar, Rajiv Raman

**Affiliations:** 1 Department of Computer Science, Birla Institute of Technology and Science Pilani, Pilani, India; 2 Sankara Nethralaya, Chennai, India; University of Manitoba, CANADA

## Abstract

Myopic Choroidal neovascularization (mCNV) is one of the most common vision-threatening com- plications of pathological myopia among many retinal diseases. Optical Coherence Tomography Angiography (OCTA) is an emerging newer non-invasive imaging technique and is recently being included in the investigation and treatment of mCNV. However, there exists no standard tool for time-efficient and dependable analysis of OCTA images of mCNV. In this study, we propose a customizable ImageJ macro that automates the OCTA image processing and lets users measure nine mCNV biomarkers. We developed a three-stage image processing pipeline to process the OCTA images using the macro. The images were first manually delineated, and then denoised using a Gaussian Filter. This was followed by the application of the Frangi filter and Local Adaptive thresholding. Finally, skeletonized images were obtained using the Mexican Hat filter. Nine vascular biomarkers including Junction Density, Vessel Diameter, and Fractal Dimension were then computed from the skeletonized images. The macro was tested on a 26 OCTA image dataset for all biomarkers. Two trends emerged in the computed biomarker values. First, the lesion-size dependent parameters (mCNV Area (mm^2^) Mean = 0.65, SD = 0.46) showed high variation, whereas normalized parameters (Junction Density(n/mm): Mean = 10.24, SD = 0.63) were uniform throughout the dataset. The computed values were consistent with manual measurements within existing literature. The results illustrate our ImageJ macro to be a convenient alternative for manual OCTA image processing, including provisions for batch processing and parameter customization, providing a systematic, reliable analysis of mCNV.

## Introduction

Myopic Choroidal neovascularization (mCNV) is usually of type-2 CNV that distorts macular anatomy and is a significant vision-threatening complication of pathologic myopia [1]. A population-based study reported that the mCNV was prevalent in 5–11% of individuals with pathologic myopia [2]. Several modalities such as Fluorescein Angiography (FFA), Spectral Domain Optical Coherence Tomography (SD-OCT), and Optical Coherence Tomography Angiography (OCTA) help in the diagnosis of this condition [3]. FFA is considered gold standard investigation to confirm CNVs which delineates the abnormal vasculature originated under the retinal layers.

OCTA is an emerging and newer non-invasive modality to obtain retinal cross sectional scans which could have an advantage of quantitative analysis to monitor CNV in various macular conditions. Cheng et al. [4] reported mCNV area and flow area as indicators of the effectiveness of intravitreal therapy with Anti-VEGFs (Anti Vascular Endothelial Growth Factor). Wang et al. [1] developed on this and discussed more OCTA biomarkers, such as fractal dimension, junction density, and concluded vessel junctions to be the most predictive biomarker in patients undergoing anti-VEGF treatment. Li et al. [5] found branching to be the most sensitive criterion to determine further treatment. They developed a novel procedure combining OCTA and vascular branching to evaluate neovascular activity in mCNV.

All the studies mentioned above developed their own custom programs to process the OCTA images. Software such as MATLAB and ImageJ (Fiji) were commonly used for image processing. Although these have been effective in measuring the biomarkers, there seems to exist no standard tool to aid in the quantitative analysis of OCTA images of mCNV. We aim to fill in this gap by developing an ImageJ macro for the exact purpose of measuring biomarkers from manually segmented OCTA images obtained by commercial SD-OCT systems.

**Fig 1:**
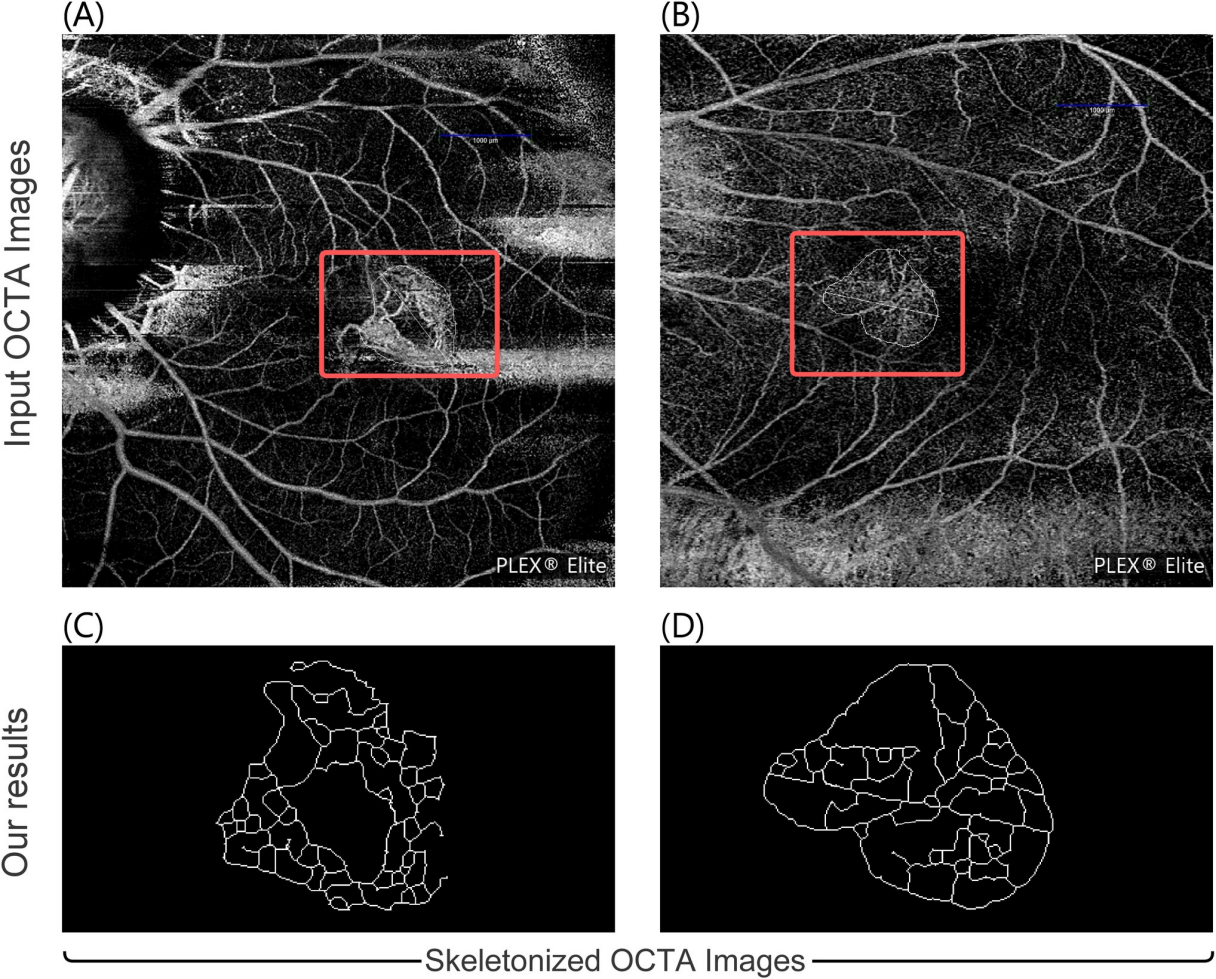
Skeletonization and quantitative analysis of OCTA images. We present an ImageJ macro that processes OCTA images of mCNV (A), (B). Our work enables users to perform quantitative analysis on skeletonized images (C), (D) of active mCNV lesions using nine computed biomarkers.

In the present study, we propose an ImageJ (a Java-based image processing program developed by the National Institute of Health, USA) macro that allows users to measure nine mCNV parameters from OCTA images. We built upon the algorithm proposed by Wang et al. [1], for mCNV image processing, and applied the Mexican Hat Filter before skeletonization. While [1] focusses on the quantitative analysis of the morphological changes in patients with mCNV, our main goal is to provide a standard software tool to automate this process for the analysis of mCNV lesions. Our macro grants users finer control over the image processing parameters and presents the extracted parameters for a batch of preprocessed OCTA images in tabular form. It also allows users to save intermediate stages of the mCNV images for further analysis. Fig 1 shows the sample input (OCTA images of mCNV) and outputs of our macro—skeletonized images.

The remainder of this paper is organized as follows. We begin by describing the acquisition of OCTA image, and the nine parameters we consider. Next, we discuss the image processing pipeline and its various stages. This is followed by our results and comparison to a similar study. Finally, we conclude with the limitations of this study, and further research paths.

## Materials and methods

### OCTA image acquisition

During the development phase, we tested the macro on a dataset of 26 expert-labeled OCTA images from 24 patients. This set of OCTA images was obtained through a research collaboration with Vitreoretina department, Sankara Nethralaya, Chennai, India. The inclusion and exclusion criteria for the study are shown in Table 1. This research was approved by the ethics committee (IRB number- 805-2019-P) at Sankara Nethralaya and have been conducted according to the principles expressed in the Declaration of Helsinki. This being a retrospective study, the IRB waived the requirement for informed consent. The OCTA images had a resolution of 1024x1024, at a scale of 170 pixels/mm, and were randomly collected retrospectively from patients with mCNV in different stages of treatment.

**Table 1:** Inclusion and exclusion criteria for the mCNV study.

Inclusion criteria	Exclusion Criteria
• Adults between the ages 18 and 65	• Age-related macular degeneration
• Dioptric power of more than -6D	• Diabetic Macular Edema
• Axial Length greater than 26mm	• Retinal vascular diseases
• Post-Lasik and Pseudophakic patients who are clinically suspected of myopic maculopathy on fundus examination.	• Retinal detachment and any maculopathy not related to myopia

### Quantitative evaluation of mCNV images

Our macro script to process can analyze batches of OCTA images and is written in the ImageJ 1.x Macro language [6]. ImageJ is a powerful Java-based image processing program developed by the National Institute of Health, USA. It is specifically designed for image analysis tasks in biological sciences, as described in the existing literature. Using ImageJ analysis tools, the macro we developed measures the following nine OCTA metrics:

1. mCNV Area (mm2): A lesion size biomarker that indicates the total size of the active mCNV lesion.
2. Vessel Area (mm2): A vessel size biomarker that indicates the size of the vessel components with flow signals in the lesion.
3. Vessel Density: A vascular biomarker defined as the ratio of the area occupied by vessels to the entire mCNV lesion area.
4. Vessel Length (mm): A vascular biomarker that indicates the total neovascular length of the CNV lesion.
5. Vessel Diameter (*μ*m): A vascular biomarker that indicates the average vessel caliber of the mCNV.
6. Vessel Junctions: A vascular biomarker defined as the number of points of vascular connection, indicating internal branching in the mCNV network.
7. Junction Density (n/mm): A biomarker, defined as the ratio of the number of junctions to the total neovascular length, indicating branching complexity.
8. Fractal Dimension: A biomarker that represents the complexity and the space-filling capacity of the vessel branching pattern.
9. Vessel Tortuosity: A morphologic biomarker that quantifies the microtortuosity of the CNV. Smaller values indicate ‘straighter’ vessels.

### Method overview

The macro uses expert-labeled retinal OCTA images as the input. We use an algorithm based on one proposed by Wang et al. [1]. The OCTA images must first be preprocessed by manual delineation. The pipeline follows a branched structure (Fig 1). In the first branch, a Gaussian Blur filter is used to denoise the images. This is followed by a Hessian-based Frangi filter to detect vessels. Finally, a binary image is obtained using Auto Local Median Thresholding. At this stage, the values of Vessel Area, mCNV Area, and Vessel Density are calculated. In the second branch, Mexican Hat Filter [7] is applied to the delineated OCTA images, followed by Skeletonization. The skeletonized image is used to calculate the values of Vessel Length, Junctions, Fractal Dimension and Torutosity. Vessel Diameter and Junction density are derived from the previously calculated parameters.

### Overall pipeline structure

**Fig 2:**
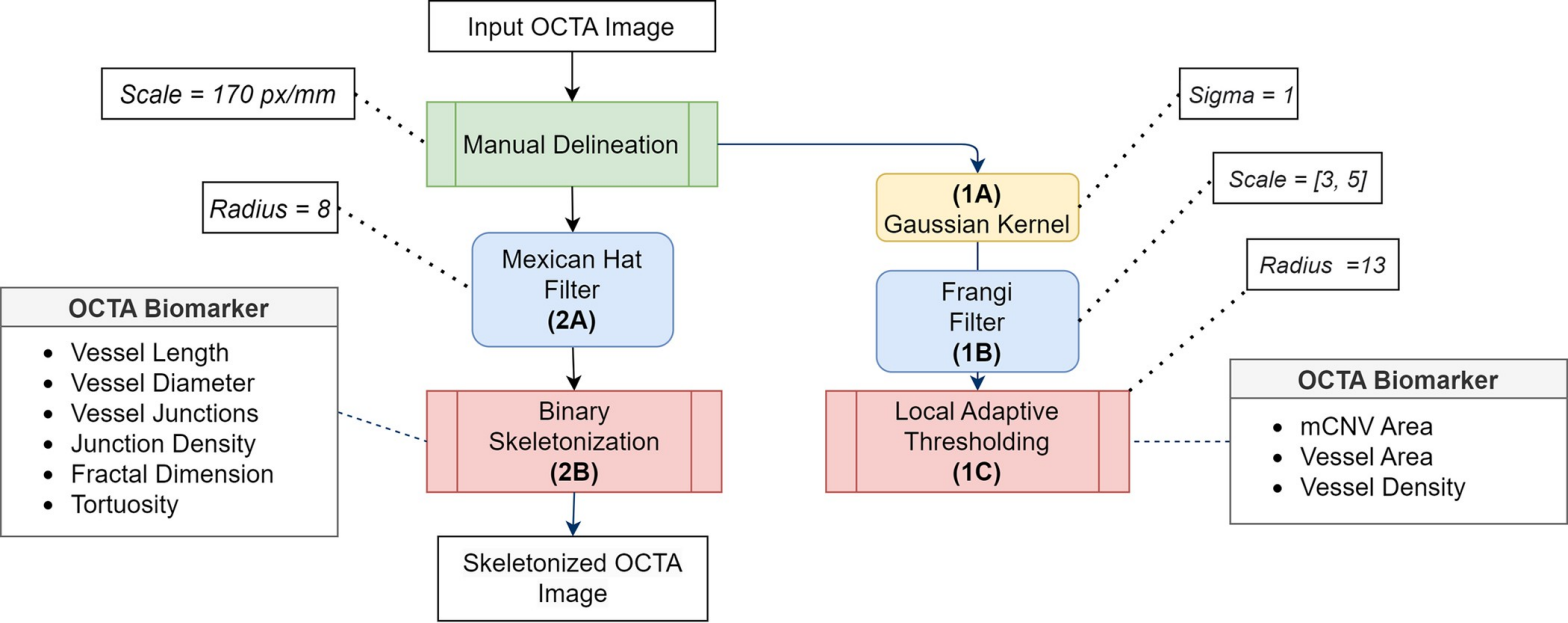
The OCTA image processing pipeline and its various stages. Our algorithm takes manually cropped OCTA images as input. The pipeline follows a branched structure. In Branch 1, we denoise the image (1A), apply the Frangi filter (1B), and use Median Thresholding (1C) to measure area-related biomarkers. In Branch 2, we apply the Mexican Hat filter (2A) and Binary Skeletonization (2B) to measure vascular biomarkers like junctions, diameter, fractal dimension, and tortuosity.

Fig 2 shows the overall image processing pipeline of the application. The various stages are detailed below.

#### Preprocessing

Before entering the pipeline, the expert-labeled OCTA images must be manually delineated to ensure that the vessel biomarkers are measured only in the active mCNV lesion. The cropped images are converted to 8-bit grayscale as a precondition for the Skeletonization stage (2C) further down the pipeline. The mCNV lesion area is measured from the cropped OCTA image. The brightness and contrast of the cropped image are adjusted to reduce noise.

#### Gaussian Kernel (1A)

The cropped images are further denoised using a Gaussian Blur filter with a radius of decay as 1. This filter uses convolution with a Gaussian function (1) for smoothing [8]. The quality of single OCTA images of mCNV is poorer than averaged images, so denoising is beneficial [9] for the Frangi filter stage (1B).


$$G(x,y)=\frac{1}{2\pi \sigma -x^{2}+y^{2}e\;2\sigma^{2}}$$ (1)

#### Hessian Frangi Filter (1B)

The denoised images are passed through a Hessian-based Frangi vesselness filter (using the ImageJ Frangi plugin [10]). This filter uses the eigenvectors of the Hessian to compute the likeliness of the 2D image to contain vessels (Frangi et al. [11]). The Frangi vesselness filter is crucial for the Thresholding stage (1C).

#### Auto local thresholding (1C)

The 8-bit images are binarized through local adaptive thresholding (using the ImageJ Auto Local Threshold plugin [12]). ‘Local’ here means that the threshold is computed for each pixel within a window of a certain radius (= 8) around it. The Median method selects the threshold as the median of the local greyscale distribution. The median method was consistently produced better segmentation results than other methods such as the Otsu algorithm [13]. The area statistics of the binary image are used to find the Vessel Area. The Vessel Density biomarker is calculated using the values of Vessel Area and mCNV Area.

#### Mexican Hat Filter (2A)

This branch of the pipeline is used to produce skeletonized binary OCTA images. The cropped images are passed through the Mexican hat Filter (Mexican hat filter plugin [14]). The filter is applied over a neighborhood of radius 13 for each pixel (radius value found empirically). This filter uses convolution with the Ricker wavelet function (2) and has proven to be effective for feature detection [7]. The Mexican Hat Filter stage is the precursor to Skeletonization.


$$\psi (x,y)=\frac{1}{\pi \sigma^{4}}\left(1-(\frac{x^{2}+y^{2}}{2\sigma^{2}})\right)e^{-\frac{x^{2}+y^{2}}{2\sigma^{2}}}$$ (2)

#### Binary Skeletonization (2B)

Finally, The 8-bit OCTA images are skeletonized (internally implemented using the Binary Thinning Algorithm (Homann et al. [15]])). The skeletonized images provide great insight into the vascular activity of the mCNV lesion. The ‘AnalyzeSkeleton’ ImageJ plugin [16] provides a detailed statistical analysis of the branching structure. Furthermore, it is used to prune disconnected vertices and branches that end in end-points. The results are used to calculate the Vessel Length, Tortuosity, and number of Junctions. Ratios such as Junction Density and Vessel Diameter are computed using the skeletonized image as well. The vessel complexity is quantified using Fractal Dimension, which is measured by the box-counting method (3). At this stage, all nine biomarkers have been established.

**Fig 3:**
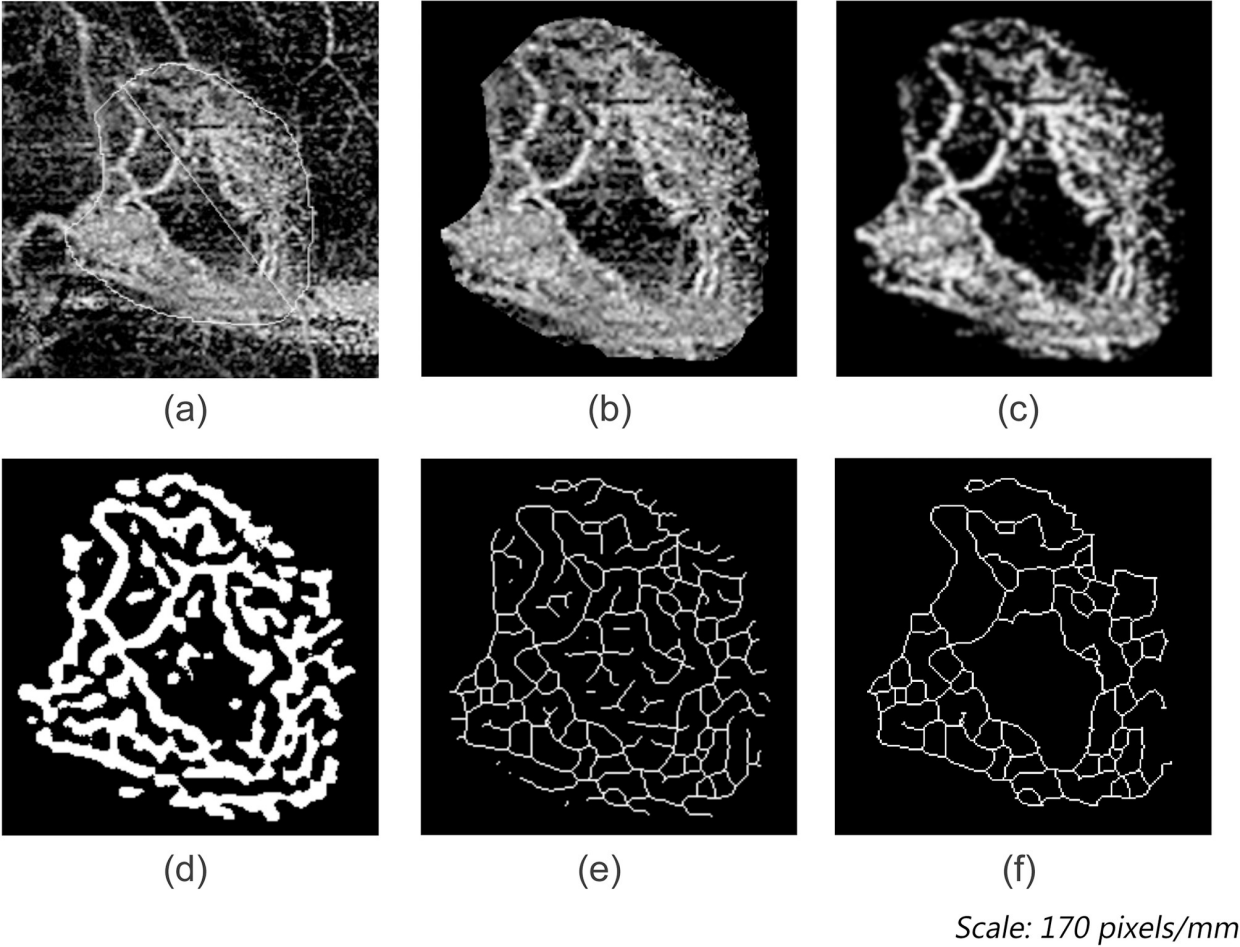
Intermediate stages of the OCTA image as it travels down the pipeline. (a) Input OCTA image showing the active mCNV lesion. (b) Manually delineated OCTA image. mCNV area is measured by counting the pixels within the contour. (c) The Gaussian kernel and Contrast adjustment were used for smoothing and denoising. (d) The Frangi vesselness filter and Mexican Hat filter were used to calculate Vessel Area and Vessel Density. (e) Binary skeletonization of the OCTA image using ImageJ. (f) The tagged skeleton was used to calculate the Junctions, Vessel Diameter, Tortuosity, and Fractal Dimension.

Fig 3 shows all the intermediate pipeline stages on a sample OCTA image, starting with the input image and ending with the tagged skeleton.


$$D_{box}=lim_{s\to 0}\;logN(s)\;logs$$ (3)

### Program description

The macro can be launched in Fiji without installation. Once the macro is downloaded, it can be opened by using the Macro option in the Plugins window or by simply dragging and dropping the script onto an active ImageJ window. The following choices are offered to the user, on launching the script.

Type of Analysis: The user can input the number of images they want to analyze. A checkbox enables the user to save all the intermediate image processing steps. The user can choose the input image file format, which is set to TIFF by default. The user also selects the target input and output folders for the input images, the output CSV file, and the intermediate pipeline stages. Input Images: It is highly recommended that the input OCTA images be manually delineated in ImageJ, to achieve accurate results. This can be achieved using any of ImageJ’s in-built selection tools, such as Polygon, Freehand, etc., followed by clearing the image area outside the selection.

Customizable Parameters: The user can then adjust the values of parameters in various pipeline stages. The sigma of the Gaussian used in stage 1A can be adjusted depending on the amount of denoising needed. The user can also set the pixel radii for the Local Adaptive Thresholding in Stage 1C and the Mexican Hat filter in Stage 2A, based on the mCNV lesion in the input images. Finally, the user can set the pixel scale (per 1000 μm) for all the subsequent measurements.

Once the script processes the entire batch, a CSV file containing the computed values of all 9 OCTA biomarkers is saved in the output directory. Additionally, if the user checks the “Save Pipeline Stages?” checkbox, the intermediate images (5 per input image) are stored in the specified output directory.

System Requirements: The software requirements are the same as those of Fiji (ImageJ), currently supported on:

- Windows XP, Vista, 7, 8 and 10
- Mac OS X 10.8 “Mountain Lion” or later
- Linux on amd64 and x86 architectures

However, the availability of Java 8 runtime is the only software pre-requisite.

### Mode of availability

The ImageJ macro is open-access software and can be found (along with brief instructions) in the Supporting Information. The only additional dependency is the Mexican Hat Filter, which can be found and installed from https://imagej.nih.gov/ij/plugins/ mexican-hat/index.html.

## Results

The primary purpose of the macro is to perform quantitative analysis on OCTA images of mCNV and obtain reliable measurements of nine biomarkers. We tested the macro on a dataset of 26 manually-cropped expert-labeled OCTA images from 24 patients. (courtesy of Sankara Nethralaya, Chennai, India). The total runtime of our program (excluding preprocessing) is 57.31 seconds, significantly less than the time required manual quantitative analysis of these biomarkers which may take upto thirty minutes.

In our results (Table 2), we observed that parameters like mCNV Area (0.65 ± 0.4 mm2), Vessel Area (0.31 ± 0.20 mm2), Vessel Junctions (59.88 ± 42.81), and Vessel Length (10.24 ± 6.77 mm) had high standard deviation in comparison to their mean values, which indicates a large amount of variation across different images. On the other hand, metrics such as Tortuosity (1.12 ± 0.02), Fractal Dimension (1.34 ± 0.05), Vessel Diameter (30.02 ± 1.44 *μ*m), Junction Density (5.64 ± 0.63 n/mm), and Vessel Density (0.49 ± 0.02) had low standard deviation, indicating that values were clustered around the central value.

**Table 2:** Statistical analysis of OCTA biomarkers on the 26-image dataset.

OCTA Biomarkers	Mean	SD	Min	Max
mCNV area, mm2	0.65	0.46	0.16	1.95
Vessel Area, mm2	0.31	0.20	0.08	0.86
Vessel Junctions	59.88	42.8	10.00	183.00
Vessel Length, mm	10.24	6.77	2.62	29.32
Fractal Dimension	1.34	0.05	1.19	1.41
Tortuosity	1.12	0.02	1.10	1.19
Vessel Density	0.49	0.02	0.44	0.51
Junction Density, n/mm	5.64	0.63	3.82	6.58
Vessel Diameter, *μ*m	30.02	1.44	28.15	33.56

We compared the reliability of our results against a similar analysis by Wang et al. [1] (dataset of 31 OCTA images from 29 patients’ eyes (retinae)). The statistical analysis for both studies is shown in Table 3. Our measurements for most parameters were consistent with those in existing literature (Table 3). Any differences in the mean or standard deviation were likely due to variation in the datasets. The nine OCTA biomarkers computed by the macro were within the range of expected values obtained manually.

**Table 3:** Comparison of our results (26 images) with a similar study on mCNV (Wang et al. [1], 31 images).

OCTA Biomarkers	Our Results		Results by Wang et al.	
	Mean	SD	Mean	SD
mCNV area, mm2	0.65	0.46	0.40	0.52
Vessel Area, mm2	0.31	0.20	0.20	0.20
Vessel Junctions	59.88	42.81	49.36	47.43
Vessel Length, mm	10.24	6.77	6.96	7.92
Fractal Dimension	1.34	0.05	1.08	0.15
Tortuosity	1.12	0.02	1.26	0.07
Vessel Density	0.49	0.02	0.55	0.09
Junction Density, n/mm	5.64	0.63	7.52	1.65
Vessel Dimaeter, *μ*m	30.02	1.41	31.11	3.78

## Discussion

The ImageJ macro designed in the present study follows a branched pipeline strcuture and builds upon the algorithm proposed by Wang et al. [1] with the use of the Mexican Hat Filter for skeletonization. It was able to automate the computation of 9 biomarkers such as mCNV Area, Vessel Density and Junctions, Fractal Dimension etc. from OCTA images of mCNV. The macro was deployed on 26 OCTA images (1024x1024) obtained through a research collaboration with the Vitreoretina department, Sankara Nethralaya, Chennai, India.

The measurements of OCTA biomarkers obtained with the macro are valid, as they’re within the range of expected values obtained manually. The trends that emerged in the biomarkers measured in the present study were: (1) High Standard deviation in mCNV Area, Vessel Area, Vessel Junctions, and Vessel Length; (2) Low Standard deviation in Tortuosity, Fractal Dimension, Vessel Diameter,

Junction Density, and Vessel Density. The high standard deviation in the values of the former parameters can be explained by the fact that they are lesion-size dependent and that the dataset contained differently sized mCNV lesions, ranging from 0.156 mm2 (14 junctions) to 1.951 mm2 (183 junctions). Conversely, the low variation in parameters like Vessel Density and Junction Density can be attributed to them being normalized metrics, making them size-independent. Similarly, our results also indicate that Tortuosity and Fractal Dimension have low standard deviations due to them being normalized metrics, as well as similarities in vascular branching patterns across the OCTA images [17].

These results are consistent with several related studies. Li et al. [5] also observed high variability in the mCNV area (0.62 ± 0.58 mm2) in their study on a dataset of 41 eyes with mCNV. Among the parameters with low variability, Mao JB et al. [18] reported deep vessel density as 46.21 ± 5.17%, remarkably similar to our value of 48.5 ± 2.1%. Similarly, Yao X et al. [19] reported a mean difference of -1.3 *μ*m for vessels shorter than 45 μm in OCTA scans. Our values of vessel diameter 30.02 ± 1.41 *μ*m substantiate this finding as well.

However, we must note the limitations of this study. As mCNV is a relatively rare complication of pathological myopia, and the procedure to obtain OCTA images is tedious the number of OCTA images in our dataset is relatively small (26 images from 24 patients). This could be a reason why some biomarkers like Tortuosity and Fractal Dimension showed minimal variation and were nearly constant across all 26 images. Secondly, manual delineation of the mCNV area is imperative for accurate measurements from the macro. This may limit the scalability of the macro and its use on a larger batch of images. One of the important limitations is difficulty in acquiring the images in myopic eyes due to long axial length and the presence of posterior staphyloma. This causes stretched and less clear images, which in turn pose a challenge to manually segment the mCNV area. Therefore, the accuracy of the biomarker measurements may be compromised due to artifacts in the skeletonized images. These artifacts are likely due to the CNV boundaries present in the input OCTA images (annotated by experts).

Our results are promising and provide researchers with a customizable, efficient, and reliable method to perform quantitative analysis on OCTA images of mCNV. This opens up an exciting new avenue of study focused on image processing techniques to analyze retinal images. Future work in this domain could take a number of research paths, exploring different thresholding techniques, measuring even more OCTA biomarkers, or using other methods such as the Haursdoff Method to compute parameters like the Fractal Dimension of mCNV. Another promising direction is the automation of the preprocessing steps i.e. manual delineation of OCTA images using. Since the main focus, is on the quantitative analysis, we leave the automation of this as a problem for future studies.

## Conclusion

This paper discusses the problem of automating the quantitative analysis of OCTA images of mCNV, a serious complication of severe pathological myopia. We propose an image-processing pipeline that measures nine vascular biomarkers by processing batch of OCTA images and returning the corresponding skeletonized images of the mCNV lesion. We test the macro on a dataset of 26 OCTA images, provided by a tertiary care ophthalmic center. We observe that vascular parameters like ‘mCNV Area’ vary widely across the images depending on the patient, whereas normalized parameters like ‘Tortuosity’ and ‘Vessel Density’ show far less variation. Our results are not only consistent with existing studies on mCNV, but our automated method significantly expedites such quantitative analyses of mCNV. Future studies could consider expand to more parameters or adopt different techniques, such as the Haursdoff Method for current parameters like Fractal Dimension. A promising research direction is the automation of the preprocessing steps, to completely automate the analysis of mCNV lesions.

## Supporting information

S1 File (PDF)

S2 File (IJM)

S3 File (XLSX)
