# Supplementary material for: An ImageJ macro tool for OCTA-based quantitative analysis of Myopic Choroidal neovascularization
Source: PLoS One. 2023 Apr 21;18(4):e0283929. doi: 10.1371/journal.pone.0283929 (PMC10120933; doi:10.1371/journal.pone.0283929)

An ImageJ macro tool for OCTA-based Quantitative Analysis of retinal mCNV

This is an ImageJ macro that allows users to automate the batch processing of retinal OCTA images of mCNV and measure nine biomarkers to characterize the lesion and vascular activity.

Description

This ImageJ macro allows users to automate the processing of OCTA images of retinae with mCNV. The macro's image processing pipeline uses Gaussian blur filter, Frangi Vesselness filter, Local Median thresholding and the Mexican Hat filter. The macro allows users to adjust the input parameters, number of images, and the scale. Users can then measure nine biomarkers including mCNV area, vessel area, vessel density, vessel diameter, vessel junctions, junction density, fractal dimension and vessel tortuosity.

Installation and Use

Once you download the ijm file, simply drag the file onto the ImageJ (Fiji) icon. With the mCNV image open, enter the desired parameters and select the input and output directories to save the results. If you wish to save images of the intermediate processing steps, check the "Save Pipeline Stages" option.

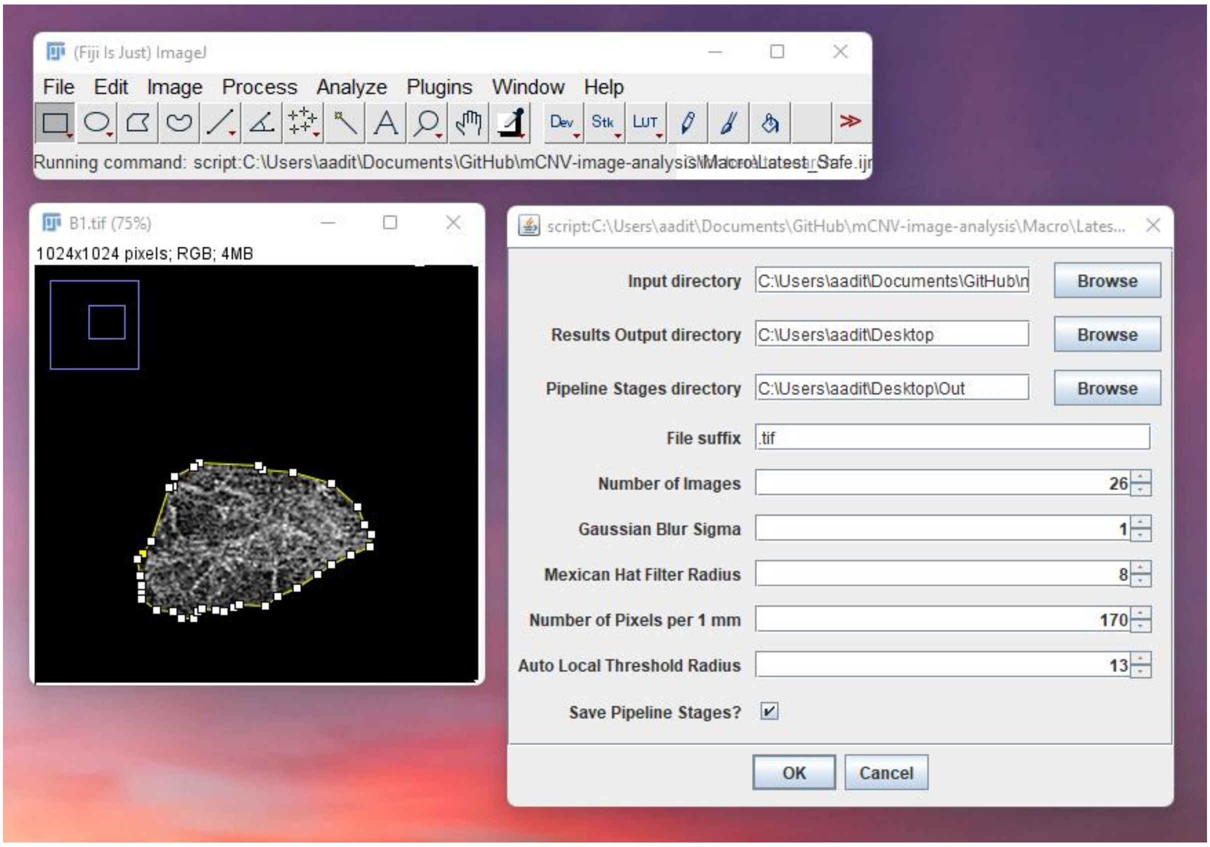

Supplement: S1 File — (PDF) [file pone.0283929.s001.pdf]
